# The Second International Conference “Genetics of Aging and Longevity”

**DOI:** 10.18632/aging.100458

**Published:** 2012-05-31

**Authors:** Vladimir N. Anisimov, Andrzej Bartke, Nir Barzilai, Mikhail A. Batin, Mikhail V. Blagosklonny, Holly Brown-Borg, Yelena Budovskaya, Judith Campisi, Bertrand Friguet, Vadim Fraifeld, Claudio Franceschi, David Gems, Vadim Gladyshev, Vera Gorbunova, Andrei V. Gudkov, Brian Kennedy, Maria Konovalenko, Brian Kraemer, Alexey Moskalev, Isabelle Petropoulos, Elena Pasyukova, Suresh Rattan, Blanka Rogina, Andrei Seluanov, Mikhail Shaposhnikov, Robert Shmookler Reis, Nektarios Tavernarakis, Jan Vijg, Anatoli Yashin, Piotr Zimniak

**Affiliations:** ^1^ N.N. Petrov Research Institute of Oncology, St-Petersburg, Russia; ^2^ Southern Illinois University School of Medicine, Springfield, USA; ^3^ Albert Einstein College of Medicine, Bronx, USA; ^4^ Science for Life Extension Foundation, Moscow, Russia; ^5^ Roswell Park Cancer Institute, Buffalo, NY, USA; ^6^ University of North Dakota School of Medicine & Health Sciences, Grand Forks, USA; ^7^ University of Amsterdam, Amsterdam, Netherlands; ^8^ Buck Institute for Research on Aging, Novato, California, USA; ^9^ University Pierre and Marie Curie, Paris, France; ^10^ Ben-Gurion University of the Negev, Beer Sheva, Israel; ^11^ University of Bologna, Bologna, Italy; ^12^ University College London, London, UK; ^13^ Harvard Medical School, Boston, USA; ^14^ University of Rochester, Rochester, USA; ^15^ University of Washington, Seattle, USA; ^16^ Institute of Biology, Komi Science Center of RAS, Syktyvkar, Russia; ^17^ Institute of Molecular Genetics of RAS, Moscow, Russia; ^18^ Aarhus University, Aarhus, Dutch; ^19^ University of Connecticut Health Center, Farmington, USA; ^20^ University of Arkansas for Medical Sciences, Little Rock, USA; ^21^ University of Crete, Heraklion, Greece; ^22^ Duke University, Durham, USA

The ongoing revolution in aging research was manifested by the Second International Conference “Genetics of Aging and Longevity” (Moscow, April 22-25, 2012). It was organized by the Science for Life Extension Foundation in collaboration with the Gerontological Society of the Russian Academy of Sciences and the International Association of Gerontology and Geriatrics (European Region). More than 250 gerontologists and and other researchers in the area of aging from 27 countries (Austria, Armenia, Belarus, Brazil, Germany, Greece, Denmark, Israel, India, Iran, Italy, Kazakhstan, Canada, Latvia, Lithuania, Nigeria, Netherlands, Poland, Portugal, Puerto Rico, Russia, United Kingdom, USA, Ukraine, France, South Korea, Japan) gathered to discuss current problems of genetics, life expectancy, and aging mechanisms. Among these scientists were invited speakers Vladimir Anisimov, Vladislav Baranov, Andrzej Bartke, Nir Barzilai, Mikhail Blagosklonny, Holly Brown-Borg, Judith Campisi, Vadim Fraifeld, Claudio Franceschi, David Gems, Vadim Gladyshev, Vera Gorbunova, Andrei Gudkov, Valter Longo, Brian Kennedy, Brian Kraemer, Kyung-Jin Min, Richard Morimoto, Alexey Moskalev, Thomas Perls, Suresh Rattan, Blanka Rogina, Andrei Seluanov, Robert Shmookler Reis, Yousin Suh, Nektarios Tavernarakis, Jan Vijg, Anatoli Yashin, and Piotr Zimniak. The Conference goal was to identify the most promising areas of genetics, life expectancy, and aging, including: the search for longevity genes; the search for pharmacological agents that slow aging; the identification of biological age markers; and the identification of mechanisms by which the environment influences the aging rate.

**Judith Campisi** (Buck Institute for Research on Aging, USA) presented “Cancer and Aging: Rival Demons?” Cellular senescence is a potent tumor suppressive mechanism that halts the proliferation of cells at risk for neoplastic transformation. Senescent cells accumulate with age and at sites of age-associated pathology in many tissues. There is mounting evidence that senescent cells are not mere bystanders; rather, they contribute to aging phenotypes and age-related disease and thus may be an example of evolutionary antagonistic pleiotropy. One strong candidate is the senescence-associated secretory phenotype (SASP). The SASP entails the transcriptional upregulation and secretion of numerous cytokines, growth factors and proteases that can have profound paracrine effects. The SASP can disrupt the structure and function of normal tissues. It can also, ironically, provoke malignant phenotypes in premalignant cells. Thus, the SASP may be the mechanism by which senescent cells drive both the degenerative and hyperplastic pathologies of aging. What controls the SASP? They identified three signaling pathways that are important positive regulators of the SASP. These are the DNA damage response pathway, the p38MAPK-NFkB pathway, and, recently, the mTOR pathway. mTOR signaling is of particular interest because it ties the SASP to an evolutionary conserved pathway that regulates longevity in diverse species, ranging from yeast to mice. Why did complex organisms evolve a senescence response and a SASP? There is little doubt that the senescence growth arrest evolved to suppress the development of cancer. The SASP, by contrast, may have evolved to promote tissue repair. Results from a new mouse model developed by the group strongly indicate that this is indeed the case for skin wounds. Thus, the senescence response subserves three processes: tumor suppression, tissue repair, and age-related pathology, including, ironically, cancer.

**Brian Kennedy** (Buck Insitute for Research on Aging, USA) presented “The TOR pathway and aging – insights from genetic screens”. Evidence from invertebrate model organisms has indicated that many pathways modulating aging are conserved. Among the most conserved is the TOR pathway, which acts as a cellular monitor of nutrient levels in the environment. Reduced TOR signaling promotes stress resistance and enhanced longevity in yeast, worms, flies and mice – the most studied aging model organisms. Current work is aimed at understanding the pathways downstream of TOR that contribute to aging. Rapamycin, a specific mTOR inhibitor confers robust extension of lifespan and healthspan in mice, offering promise that it will be possible to develop therapeutic agents aimed at extending healthspan. Currently, derivative of rapamycin are being tested in the Kennedy lab to enhance protection against age-related diseases while simultaneously reducing side effects. Increasingly, it seems possible that it will be possible to intervene in the aging process and the TOR pathway is among the most promising targets.

**Mikhail V. Blagosklonny** (Roswell Park Cancer Institute, Buffalo, USA) presented a report “mTOR from cellular senescence to organismal aging”. The nutrient-sensing TOR (Target of Rapamycin) pathway is involved in cellular and organismal aging. Pharmacological interventions that inhibit this pathway increase life span in yeast, nematode, Drosophila and rodents. In a number of experiments, rapamycin suppressed and slowed down cellular senescence of human cells. The mTOR inhibitor rapamycin prevents age-related weight gain, decreases rate of aging, increases life span and decreases carcinogenesis in mice.

**David Gems** (University College London, U.K.) presented “Does hyperfunction cause aging in C. elegans?” Many recent studies have failed to support the view that oxidative damage causes aging in C. elegans. One possibility is that the stochastic damage-somatic maintenance paradigm of aging is incorrect. An alternative proposed mechanism involves quasi-programmed hyperfunction (manifested e.g. as hypertrophy). There are many signs of hypertrophy in aging C. elegans, e.g. yolk over-production and cuticular thickening. Quasi-programmed apoptosis simultaneously causes gonadal disintegration (atrophy) and oocyte gigantism (hypertrophy) in aging C. elegans hermaphrodites. Hermaphrodite specificity of physiological germline apoptosis accounts for sex differences in pathology of aging in the gonad. The hyperfunction theory provides a full and simple account for a major age-related pathology in C. elegans. Hyperfunction is a viable alternative to molecular damage as a central mechanism of aging in C. elegans.

**Vladimir Anisimov** (N.N. Petrov Research Institute of Oncology, Russia) in his presentation “Do we really have a medicine against aging?” showed results of experiments on effects of antidiabetic biguanides and rapamycin on biomarkers of aging, life span, spontaneous and chemically-induced carcinogenesis in outbred, inbred and transgenic HER-2/neu mice and in rats. The mTOR inhibitor rapamycin prevents age-related weight gain, decreases rate of aging, increases life span and decreases carcinogenesis in transgenic HER-2/neu cancer-prone mice. Rapamycin dramatically delayed tumors onset, decreased a number of tumors per animal and tumor size. Lifelong administration of rapamycin extends lifespan in female 129/Sv mice characterized by normal mean lifespan of 2 years. Importantly, rapamycin was administrated inter-mittently (every other 2 weeks) starting from the age of 2 months. Rapamycin inhibited age-related weight gain, decreased aging rate, increased lifespan (especially in the last survivors) and delayed spontaneous cancer. 22.9% of rapamycin-treated mice survived the age of death of the last mouse in control group. Treatment of female outbred SHR mice with metformin started at the age of 3 months increased mean life span by 14% and maximum life span by 1 month. Same treatment started at the age of 9 months insignificantly increased mean life span by 6%, whereas treatment started at the age of 15 months failed to increase life span. When started at the age of 3 and 9 months, metformin delayed time of the first tumor detection by 22% and 25%, correspondingly. Thus, in female SHR mice, metformin slowed down aging and somewhat postponed appearance of tumors when started at the young and middle but not at the old age. Yet, metformin improved reproductive function when started at any age. The chronic treatment of inbred 129/Sv mice with metformin slightly modified the food consumption but failed to influence the dynamics of body weight, decreased by 13.4% the mean life span of male mice and sligtly increased the mean life span of female mice. The treatment with metformin failed influence spontaneous tumor incidence in male 129/Sv mice and decreased by 3.5 times the incidence of malignant neoplasms in female mice. Metformin increased mean life span by 8% and mammary adenocarcinoma (MAC) latency by 13.2% (p<0.05) in transgenic HER2/neu mice. The treatment metformin inhibited the growth of transplantable HER2 mammary carcinoma in FVB/N male mice by 46% at the 45th day after transplantation (p<0.001). We also have shown that phenformin or metformin inhibits 1,2-dimethylhydrazine-induced colon carcinogenesis in rats, X-rays, N-nitrosomethylurea- or 7,12-dimethylbenz(a)anthracene-benzo(a)pyrene-induced mammary carcinomas in rats, cervicovaginal, skin and soft tissue tumorigenesis in mice. These results suggest that both metformin and rapamycin may be useful in prevention of cancer and extension of lifespan when used in rational and appropriate ages, doses and schedules.

**Andrei Gudkov's** (Roswell Park Cancer Institute, Buffalo, USA) presentation was focused on a recent discovery made in his lab of a new role of p53 as a regulator of epigenetic silencing of non-coding part of mammalian genome and deregulation of this major p53 function in cancer and aging.

**Andrzej Bartke** (Southern Illinois University School of Medicine, USA) presented “Longevity benefits of endocrine defects”. Mutations which interfere with the production of, or with actions of, growth hormone (GH) increase life expectancy in mice by as much as 60%. An important implication of this finding is that the average and maximal lifespan are not fixed characteristics of a species but can be altered by mutation of a single gene. These long-lived mutant mice remain healthy and vigorous much longer than their genetically normal siblings and are partially protected from cancer. Results available to date indicate that reduced GH signaling slows aging and extends longevity by multiple interacting mechanisms. These mechanisms include increased stress resistance, improved insulin signaling, reduced mTOR activity, alterations of metabolism and mitochondrial function, a shift of secretory profile of adipose tissue from pro- to anti-inflammatory, improved maintenance of stem cell populations and organ-specific changes in expression of numerous genes. Interestingly, it was recently reported that genetically GH resistant humans never get diabetes and never die of cancer. Moreover, people from exceptionally long-lived families have improved insulin sensitivi-ty. Improved insulin signaling and other characteristics associated with extended longevity of GH-resistant and GH-deficient mice can be safely induced in humans by physical activity and a modest reduction of food intake.

**Nir Barzilai** (Einstein College of Medicine, USA) presented “Lipid profiles and genotypes associated with exceptional longevity in humans”. We assessed phenotype and genotype of over 540 subjects with exceptional longevity and their families. The families of the longest living subjects are very distinct by their lipid profile (High HDL levels and large lipoprotein particle sizes). However no major role of lifestyle factor was noted in these subjects as the rates of overweight, smoking, physical activity where generally worse over that reported in their cohort. On the other hand, there are several genotypes that have been significant in such subjects, including those in the GH/IGF pathway, lipid metabolism, thyroid metabolism, and FOXO3A that have been confirmed in other groups. These findings indicate that the genome of subjects with exceptional longevity is significantly different than those who die at a younger age, and that this genome has been protecting them against some of the worst environmental factors.

**Alexey Moskalev** (Institute of Biology at Komi Science Center, RAS, Russia) presented “Drosophila melanogaster life extension by overexpression of the Growth Arrest and DNA Damage-45 (GADD45) gene”. The GADD45 protein family plays an important role in stress signaling and participates in the integration of cellular response to environmental and physiological factors. GADD45 proteins are involved in cell cycle control, DNA repair and demethylation, apoptosis, cell survival and senescence, and inflammatory response by complicated protein-protein interactions. Because of their pleiotropic action, a decreased indelibility of Gadd45 members may have far-reaching consequences including genome instability, accumulation of DNA damage, and disorders in cellular homeostasis - all of which may eventually contribute to the aging process and age-related disorders (promotion of tumorigenesis, immune disorders, insulin resistance and reduced responsiveness to stress). In *Drosophila melanogaster* a single D-GADD45 ortholog has been described. Our data show that overexpression of the D-GADD45 gene in the nervous system leads to a significantly increase of Drosophila lifespan without a decrease in fecundity and locomotor activity. The lifespan extension effect is more pronounced in males than in females, which agrees with the sex-dependent expression of this gene. The longevity of *D. melanogaster* with D-GADD45 overexpression is apparently due to more efficient recognition and repair of DNA damage, as the DNA comet assay showed that the spontaneous DNA damage in the larva neuroblasts is reduced with statistical significance. Although further wide-scale research is warranted, it is becoming increasingly clear that Gadd45s are highly relevant to aging, age-related diseases (ARDs) and to the control of life span, suggesting them as potential therapeutic targets in ARDs and pro-longevity interventions.

**Suresh Rattan** (Aarhus University, Denmark) presented “Ageing interventions: anti-ageing or prohealthspan?” If ageing is understood as an emergent phenotype due to the failure of homeodynamics and not due to the action of any harmful and death-causing mechanisms, it transforms our approach towards ageing interventions from “anti-ageing” to “healthy ageing”. Ageing occurs in spite of the presence of complex pathways of maintenance, repair and defence, and there is no “enemy within”. This viewpoint makes modulation of ageing different from the treatment of one or more age-related diseases. Another important implication of understanding ageing as the inefficiency and imperfections of homeodynamics is that the prospect of developing anti-ageing magic bullets must be abandoned. This also means abandoning enemy-oriented rhetoric, such as the “war against ageing”, “defeating ageing”, and “conquering ageing” etc. Instead, interventions in ageing require a “friend-oriented” approach and the use of a positive language such as maintaining health, achieving healthy ageing, successful ageing, and preserving the homeodynamics. A promising strategy to slow down ageing and prevent or delay the onset of age-related diseases is that of mild stress-induced hormesis and hormetins. Physical, nutritional and mental hormetins which initiate stress responses and strengthen the homeodynamics are potentially effective ageing modulators. As a biomedical issue, the biological process of ageing underlies all major diseases, and although the optimal treatment of every disease, irrespective of age, is a social and moral necessity, preventing the onset of age-related diseases by intervening in the basic process of ageing is the best approach for achieving healthy ageing and extending the health-span.

**Robert Shmookler Reis** (University of Arkansas for Medical Sciences & VA Medical Center, USA), studying a set of isogenic longevity mutants in *C. elegans*, discovered numerous genes for which transcript abundances provide powerful predictors of longevity. In addition, lipid characteristics (Peroxidation Index, Fatty Acid Chain Length, %MUFAs, %PUFAs) are also valuable biomarkers of adult lifespan, and are consistent with elongase & desaturase transcript levels. Proteome features, especially KINASE profiles, reflect signaling pathway activities. These biomarkers often translate to mice and/or humans. In another study of Quantitative Trait Loci (QTLs) that naturally modify longevity as different strains evolve, Dr. Shmookler Reis identified 27 highly significant loci that impact lifespan, Darwinian fitness, and/or resistance to a variety of stresses. Of these QTLs, 13 had marked effects on longevity, and provided evidence that (1.) antagonistic pleiotropy (countervailing effects of a locus on fitness vs. lifespan) is common, but not universal (seen for about a third of loci); (2.) only a few dozen genes are responsible for the evolutionary divergence of longevity among natural isolates; and (3.) gene-gene interactions are quite common, and may chiefly affect fitness or longevity; 3 interactions (of 9 observed) provided clear and significant evidence of antagonistic pleiotropy acting at the level of gene interaction. One of the longevity loci has recently been pursued to the point of identifying the gene responsible, which turned out to be a gene involved in meiotic chromosome pairing. The allele that stabilized the genome more during meiosis was associated with shorter adult lifespan and lower stress resistance, again implying a trade-off between reproductive benefits vs. post-reproductive survival.

**Jan Vijg** (Einstein College of Medicine, USA) presented “Aging of the genome”. Genomes of somatic cells are highly dynamic and accumulate a considerable number of alterations over the life span (genetic damages, mutations, epimutations). High-throughput sequencing is an effective way to comprehensively characterize the aging genome of individual somatic cells. Random genetic alteration may cause aging through an increase in transcriptional noise. Epimutations in DNA methylation profiles are frequent and well above the background of non-conversion events. The epimutation frequency tend to increase with age. Epimutation frequency may vary across the genome.

**Claudio Franceschi** (University of Bologna, Italy) presented “High throughput technologies (OMICS) as a new tool to identify biomarkers of longevity and healthy aging”. The phenotype of centenarians is unexpectedly complex being a mixture of adaptive robustness and accumulating frailty. Centenarians do have genetic risk alleles for major age-related diseases despite their remarkable capability to postpone / avoid them. They identified 4 chromosomal regions linked to longevity (14q11.2, 17q12-q22, 19p13.3-p13.11, 19q13.11-q13.32). This is the first study that detects linkage with longevity at the APOE gene locus. Longevity is associated with mtDNA haplogroup J and higher levels of C150T mutation in the Control Region (D-loop). A significant decrease in global DNA methylation levels was observed with age (higher methylation percentage in young controls compared to all other groups). Age-related loss of DNA methylation was less pronounced in centenarians offspring, than in offspring of non long-lived parents. N-glycans profiling appears to be one of the more robust biomarker of healthy aging.

**Richard Morimoto** (Northwestern Center for Genetic Medicine, USA) presented “The stress of misfolded proteins in biology, aging and disease”. Proteins are the major constituents of our cells; they comprise the molecular machines that replicate the genome, transcribe RNA, and translate other proteins. To achieve this uncoding of biological information, each constituent protein must fold into a specific three-dimensional shape. This process of protein folding is essential, and yet error-prone synthesis, genetic polymorphisms, and physiological and environmental stress leads to misfolding and the accumulation of damaged proteins. The balance between function and dysfunction is achieved by cell stress response pathways (the heat shock response and the unfolded protein response) and the proteostasis network that ensures synthesis, folding, translocation, assembly, and clearance of the proteome. During stress and aging, however, this balance is disrupted leading to collateral damage and the amplification of protein damage with enhanced risk for age-associated diseases including, cancer, immunological disease, metabolic disease, and neurodegeneration. Therefore, key to healthspan and the prevention of these diseases is an understanding of the underlying biology of protein quality control, how damaged proteins are detected, and mechanisms of the HSR and other stress responses to enhance folding and clearance of damaged molecules.

**Brian Kraemer** (University of Washington & VA Medical Center, USA) presented “Suppressing aging related proteotoxicity: targeting tau pathology in C. elegans”. Age dependent accumulation of protein aggregates is one of the neuropathologies associated with brain aging. Indeed deposition of aggregated detergent insoluble proteins in neurons is a common feature in most aging dependent neurodegenerative disorders. Abnormal deposits of human tau protein are the most common pathological hallmarks of aging related dementia disorders including Alzheimer's disease and frontotemporal dementia. Using a transgenic *C. elegans* model for human tau pathology, we have been exploring the genetic requirements for tau neurotoxicity. In transgenic *C. elegans* expressing human tau in all neurons, we observe several hallmarks of human tauopathies including altered behavior, reduced lifespan, accumulation of detergent insoluble phosphorylated tau protein and neurodegeneration. To identify genes required for tau neurotoxicity, we have utilized forward genetic screens for mutations that suppress tau neurotoxicity and associated phenotypes. We have isolated several different mutations ameliorating the toxic effects of tau. While these mutations alter a variety of processes in neurons including: autophagy, nucleo-cytoplasmic transport, RNA processing, and microtubule function, the exact mechanisms of neuroprotection remain un-clear. Nonetheless, all suppressor mutations isolated to date reduce tau aggregation and ameliorate neurodegenerative changes. We will present new work on the conservation of tau suppressor genes from worms to humans and the role these homologous human genes play in the formation of pathological tau. Likewise the potential for novel neuroprotective and life-span extending strategies will be explored.

**Vera Gorbunova** and **Andrei Seluanov** (University of Rochester, USA) presented “Aging and genome maintenance”. Genomic instability is a hallmark of aging tissues. Genomic instability may arise due to inefficient or aberrant function of DNA double-strand break (DSB) repair. DSBs are repaired by homologous recombination (HR) and nonhomologous DNA end joining (NHEJ). HR is a precise pathway, while NHEJ frequently leads to deletions or insertions at the repair site. Our studies showed that NHEJ declines approximately 3-fold in replicatively senescent cells. We also observed reduction in NHEJ efficiency in aging mice. However, the strongest changes were observed with HR, which declined 38-fold in pre-senescent cells. Expression of multiple proteins involved in HR diminished with cellular senescence. Supplementation of these proteins either individually or in combination did not rescue senescence related decline of the HR. Remarkably, overexpression of SIRT6 in pre-senescent cells strongly stimulated HR repair. These studies suggest that activation of SIRT6 may reduce age-related genomic instability. Andrei Seluanov and Vera Gorbunova (University of Rochester, USA) also presented “Longevity and anticancer mechanisms in long-lived mole rats”. They identified a novel mechanism of cancer-resistance in the naked mole rat termed early contact inhibition (ECI). Contact inhibition is a key anticancer mechanism that arrests cell division when cells reach high density. Naked mole rat cells are hypersensitive to contact inhibition and arrest proliferation at low cell density. They found that ECI requires the activity of p53 and Rb pathways and is associated with induction of p16^INK4a^. Recently they identified the extracellular matrix component that that triggers ECI. Application of this molecule in humans opens new avenues for cancer prevention and life extension.

**Sean Curran** (University of Southern California, USA) presented “A conserved starvation response mediated by non-canonical SKN-1/ Nrf2 signaling”. SKN-1/Nrf2 plays multiple essential roles in development and maintaining cellular homeostasis. We demonstrate a novel cellular mechanism utilized by SKN-1 to execute a specific and appropriate transcriptional response to changes in available nutrients leading to metabolic adaptation. We isolated the first gain-of-function(gf) alleles of skn-1, which disrupt the association of SKN-1 with the mitochondrial outer membrane protein PGAM-5. In the presence of plentiful food, the skn-1(gf) mutants perceive a state of starvation. The aberrant monitoring of cellular nutritional status leads to an altered survival response to changes in food availability. As such, skn-1(gf) mutants transcriptionally activate genes associated with metabolism, adaptation to starvation, aging, and survival. The triggered starvation response is conserved in mice with constitutively activated Nrf2. The dysregulated metabolism in these cells may contribute to the lethal phenotype and the tumorgenicity associated with activating Nrf2 mutations in mammalian somatic cells. Taken together, our findings provide a mechanism for an evolutionarily conserved metabolic axis of SKN-1/Nrf2 activation that adds further dimensions to the complexity of this pathway.

**Dr. Holly Brown-Borg** (University of North Dakota, USA) presented “Growth hormone and aging: Does DNA methylation play a role?” Growth hormone mutant mice are diminutive in size, exhibit enhanced antioxidative capacity and extended longevity when compared to their growth hormone sufficient counterparts. Many physiologic mechanisms appear to be altered in these mice that may contribute to these differences including aspects of glutathione and methionine metabolism. The atypical methionine metabolism in the Ames dwarf mice leads to differential expression of DNA methylation enzymes (Dnmt) and differential methylation. Gene expression of the Dnmt enzymes (Dnmt1, Dnmt3a) is greater in Ames dwarf mice in comparison to age-matched wild type mice (p<0.05). However, protein levels of Dnmt1 are lower while levels of Dnmt3a protein tend to be higher in dwarf compared to wild type mice. Dnmt enzyme activity levels also differ by genotype and age and may be responsible for the differences in methylation observed in an earlier array experiment. Global DNA hypomethylation was measured using repetitive DNA elements and found to differ by genotype and age. In addition to the enhanced ability to counter oxidative stress, these epigenetic changes may contribute to altered gene expression and the overall extension of health span and lifespan enjoyed by these mice.

**Bertrand Friguet** (Université Pierre et Marie Curie, France) presented “Pathways affected by oxidative proteome modifications in cellular senescence and oxidative stress”. Accumulation of oxidized proteins is a hallmark of cellular aging. Oxidized proteins, proteins modified by lipid peroxidation and glycoxidation adducts have been previously shown to accumulate in senescent human fibroblasts WI-38 (Ahmed et al., 2010, Aging Cell, 9:252-272). By using two-dimensional electrophoresis and immunoblotting coupled with mass spectrometry, proteins targeted by these modifications have been identified and found to include proteins mainly involved in protein maintenance, energy metabolism and cytosketon. A similar proteomic approach has also been used to identify oxidized protein targets in WI-38 fibroblasts undergoing stress-induced premature senescence and only a restricted set of proteins was found targeted by carbonylation. Changes in the proteome of human myoblasts during replicative senescence and upon oxidative stress have been recently analyzed. The carbonylated proteins identified either upon oxidative stress (Baraibar et al., 2011, Free Rad. Biol. Med., 51:1522-32) or during replicative senescence are involved in key cellular functions, such as carbohydrate metabolism, protein maintenance, cellular motility and homeostasis. Interestingly, almost half of the proteins identified as increasingly carbonylated were the same during replicative senescence or upon oxidative stress. Taken together, our results indicate that proteins involved in key cellular pathways are affected upon oxidative stress and cellular senescence, the impairment of which may be implicated in cellular dysfunction. Moreover, this study underscores the importance of performing proteomic analyses addressing different aspects, such as expression level and carbonylation, to have a broader view of changes affecting the cellular proteome.

**Blanka Rogina** (University of Connecticut Health Center, USA) presented “Mechanisms of Indy life extension in D. melanogaster”. Indy encodes the fly homologue of a mammalian transporter of di and tricarboxylate components of the Krebs cycle intermediates. Reduced expression of fly Indy or two of worm Indy homologs extend longevity. In flies, INDY is predominantly expressed in places where intermediary metabolism takes place, such as gut, fat body and oenocytes. It has been hypothesized that Indy mutation mimics calorie restriction and extend longevity by related mechanism. This hypothesis is supported by the similarities in physiology of calorie restricted flies with Indy mutant flies on high calorie diet, such as lower weight, egg production, levels of triglyceride, decreased starvation resistance and increased spontaneous physical activity. In addition, Indy mutant flies have similar changes in mitochondrial biogenesis as calorie restricted animals. New findings also suggest that Indy mutation preserve homeostasis in other tissues that contribute extended health and longevity in Indy mutant flies.

**Nektarios Tavernarakis** (FORTH, Medical School, University of Crete, Greece) presented “Mitochondrial energy metabolism and protein homeostasis in aging”. Regulation of protein synthesis is critical for cell growth and maintenance. Ageing in many organisms, including humans, is accompanied by marked alterations in both general and specific protein synthesis. Whether these alterations are simply a corollary of the ageing process or have a causative role in senescent decline remains unclear. A battery of protein factors facilitates the tight control of mRNA translation initiation. The eukaryotic initiation factor 4E (eIF4E), which binds the 7-methyl guanosine cap at the 5′ end of all nuclear mRNAs, is a key regulator of protein synthesis. In addition, marked alterations in cellular energy metabolism are a universal hallmark of the ageing process. The biogenesis and function of mitochondria, the energy-generating organelles in eukaryotic cells, are primary longevity determinants. Genetic or pharmacological manipulations of mitochondrial activity, profoundly affect the lifespan of diverse organisms. However, the molecular mechanisms regulating mitochondrial energy metabolism during ageing are poorly understood. Prohibitins are ubiquitous, evolutionarily conserved proteins, which form a ring-like, high molecular weight complex at the inner membrane of mitochondria. Prohibitin function has been implicated in carcinogenesis and replicative senescence. The nematode Caenorhabditis elegans offers a versatile platform in which to investigate the potential link between protein quality control, mitochondrial energy metabolism and ageing. We have found that the mitochondrial prohibitin complex promotes longevity by moderating fat metabolism and energy production in *C. elegans*. Prohibitin deficiency shortens the lifespan of otherwise wild type animals. Remarkably, knockdown of prohibitin promotes longevity under dietary restriction, in diapause mutants and in animals under metabolic stress. Depletion of prohibitin influences ATP levels, animal fat content and mitochondrial proliferation in a genetic-background- and age-specific manner. Together, these findings reveal a novel mechanism regulating mitochondrial biogenesis and function, with opposing effects on energy metabolism, fat utilization and ageing. Prohibitin may serve a similar key role in the modulation of energy metabolism during ageing in mammals. Moreover, we find that loss of a specific eIF4E isoform, IFE-2 that functions in somatic tissues, reduces protein synthesis and extends the lifespan of *C. elegans*. Knockdown of the phosphatidyl inositol kinase TOR that controls protein synthesis in response to nutrient cues further increases the longevity of ife-2 mutants. In addition, reduction of protein synthesis increases ATP availability and stress resistance Thus, signaling via eIF4E may influence ageing by moderating energy demands and augmenting stress resistance mechanisms, though regulation of protein synthesis in the soma.

**Thomas Perls** (Boston University, USA) presented “Increasing genetic influence upon exceptional longevity with older and older ages”. Working with a wide range of disciplines including statisticians, geneticists and computer scientists, has led the production of a landmark article in which a genetic model consisting of 281 genetic markers predicts with 85% accuracy whom in their sample of controls and centenarians is age 105+ years (published this January in PLoS ONE). The accuracy of the model is lower, about 60% for nonagenarians and centenarians at age 100, which supports the hypothesis that the genetic component of survival to older and older age beyond 100 gets progressively stringer.

**Olga Mustafina** (Institute of Biochemistry and Genetics, RAS, Russia) presented “Gene polymorphism and human longevity: Association studies deliver”. In men *FOXO1A* gene polymorphism (rs4943794) is possibly associated with ageing, and *FOXO3A* gene polymorphism (rs3800231) may be important for achievement of longevity.

**Elena Pasyukova** (Institute of Molecular Genetics, RAS, Russia) presented “Drosophila melanogaster lifespan is associated with genes controlling asymmetric neuroblast division”.One of the organ systems that play a major role in maintaining homeostasis and regulating aging and longevity is the nervous system. Earlier, we demonstrated that several genes participating in regulation of the nervous system development are involved in lifespan control. An insertion of the *P{GT1}* vector 300 bp downstream of the structural part of *escargot* (*esg*) that encodes an RNA polymerase II transcription factor and insertions of *P*-based vectors in the structural part of *aPRC* that encodes atypical protein kinase C were associated with increase in male and female lifespan. Both *esg* and *aPKC* are involved in regulation of a crucial step in the nervous system development, asymmetric neuroblast division (AND). We suggested that other genes interacting with *esg* and *aPKC* during AND could be important for lifespan control. *inscrutable* (*insc*) is a gene essential for AND. We demonstrated that an insertion of the *P{EPgy2}* vector in the structural part of *insc* prolonged female lifespan. Analysis of lifespan of heterozygous *esg* and *insc* double mutants allowed us to predict that these genes interact in the course of lifespan determination. Decrease of *esg* transcription was shown to be associated with lifespan increase in mutant *esg* and *insc* flies. aPKC is directly phosphorylated by GSK3β (glycogen synthase kinase 3) encoded by *shaggy* (*sgg*), which further provides AND. We demonstrated that several insertions of *P*-based vectors in the structural part of *sgg* were associated with alterations of male and female lifespan. Altogether, our recent findings indicate that genes affecting AND during early steps of development could influence longevity of *Drosophila* adults.

**Piotr Zimniak** (University Arkansas for Medical Sciences & VA Medical Center, USA) presented “The lipid peroxidation product 4-hydroxynonenal as a modulator of fat accumulation and aging”. Several 4-HNE-metabolizing enzymes and chemical 4-HNE scavengers have a longevity assurance function in C. elegans. 4-HNE may accelerate aging either by its toxicity as an electrophile or by modulating metabolism (ectopic fat accumulation?) or signaling pathways such as insulin-like signaling. The effects of 4-HNE on longevity and on fat deposition could be linked by a metabolic syndrome-like state. 4-HNE-linked mechanisms are amenable to pharmacological treatment. The oxidative stress theory of aging is not quite dead yet, but is in need of chemical refinement.

**Isabelle Petropoulos** (Université Pierre et Marie Curie, France) presented “Role of oxidized protein repair in protection against oxidative damage: a proteomic approach”. The accumulation of oxidatively modified protein is a hallmark of aging. This accumulation results, at least in part, from the increase of reactive oxygen species coming from both cellular metabolism and external factors including environment, but the efficacy of protein maintenance systems is also involved. Most organisms, from bacteria to humans, have developed a specific reductase system, the Methionine Sulfoxide Reductase (Msr), which allows the repair of oxidized methionines in proteins. The Msr system, composed of the two stereo specific enzymes: MsrA and MsrB, plays a major role in the maintenance of protein homeostasis during aging and has also been involved in cellular defences against oxidative stress, by scavenging ROS. To analyse more precisely the relationships between the Msr system, oxidative stress and oxidative protein damage, MsrA and MsrB2, a mitochondrial member of the MsrB family, have been stably overexpressed in cellular models. Msr overexpressing cells are more resistant to H2O2 cytotoxity by delaying apoptosis and protecting against necrosis (Cabreiro et al., 2008, J Biol Chem, 283: 16673-16681). Moreover, it was demonstrated that the mechanisms by which MsrB2 protects against oxidative stress include: maintenance of a lower level of intracellular ROS, prevention of oxidized protein accumulation and protection of the proteasome against oxidative stress induced inactivation. Finally, stable human embryonic kidney cell lines (HEK) where MsrA, MsrB1 or MsrB2 gene expressions are silenced by using RNAi technology, were generated. These knocked down clones have been used in 2D-DIGE experiments (2D fluorescence difference gel electrophoresis). Most of the proteins found to be differentially expressed in Msr depleted cells were identified by mass spectrometry as proteins related to redox homeostasis. Altogether, it is suggested that the Msr proteins, in addition to be oxidized protein repair enzymes, may play an important role in protein homeostasis and cellular anti-oxidant defenses.

**Mikhail Shaposhnikov** (Insttute of Biology, Komi Science Center, RAS, Russia) in his presentation “Drosophila life extension by inhibitors of PI3K, TOR kinase and NFkB” have reported that recent progress in our understanding of genetic mechanisms of aging and longevity provides an opportunity to select some enzymes as targets for pharmacological intervention into aging speed. Phosphoinositide 3-kinase (PI3K) and TOR-kinase cascades are affected in some long-lived mutants of different animals, such as nematodes and mice. NF-κB transcription factor is one of the major regulators of gene expression associated with mammalian aging. The purpose of this study was to investigate the anti-aging potential of the inhibitors of enzymes that have known association with aging and longevity. Experimental *Drosophila* imagoes were exposed to 5 μM of LY294002, 0.5 μM of wortmannin, 0.5 μM of rapamycin, and 120 μM of pyrrolidine dithiocarbamate (PDTC) during their lifetimes. We used LY294002 and wortmannin as specific PI3K inhibitors, rapamycin as the TOR-kinase inhibitor, and PDTC as the NF-κB inhibitor. LY294002 treatment of *Drosophila* imago has led to an increase of the median (by 14%) and maximum (by 16-22%) lifespan (*p*<0.001) in females and males, respective-ly. Wortmannin treatment has induced an increase of the median (by 5%) and maximum (by 39%) lifespan in males (*p*<0.001), but the lifespan differences in females were statistically insignificant (*p*>0.05). Rapamycin treatment has induced increases of median (by 5-6%) lifespan (*p*<0.01) in males and females, respectively and increase of maximum lifespan (by 33%) in females (*p*<0.01). Treatment with PDTC has increased the median (by 11-13%) and the maximum (by 11-14 %) lifespan in females and males, respectively. Thus, we have shown that the specific pharmacological inhibition of PI3K (by LY294002 and wortmannin), TOR-kinase (by rapamycin), and NF-κB transcription factor (by PDTC) prolonged the *Drosophila melanogaster* lifespan.

**Vadim Fraifeld** (Ben-Gurion University of the Negev, Israel) presented “Linking cellular senescence and age-related diseases via miRNA regulation of protein interaction networks”. The comprehensive data mining revealed over 250 genes tightly associated with cellular senescence (CS). Using systems biology tools, it was found that CS is closely interconnected with aging, longevity and age-related diseases (ARDs), either by sharing common genes and regulators (miRNAs) or by protein-protein interactions and eventually by common signaling pathways. The patterns of evolutionary conservation of CS and cancer genes showed a high degree of similarity, suggesting the co-evolution of these two phenomena. Moreover, cancer genes and microRNAs seem to stand at the crossroad between CS and ARDs. The analysis also provides the basis for new predictions: the genes common to both cancer and other ARD(s) are highly likely candidates to be involved in CS and *vice versa*. Multiple links between CS, aging, longevity and ARDs suggest a common molecular basis for all these conditions. Modulating CS may represent a potential pro-longevity and anti-ARDs strategy.

**Anatoly Yashin** (Duke University, USA) presented “Mechanisms of aging and longevity: Lessons from genetic analyses of longitudinal data”. The associations of candidate genes with lifespan detected in a number of studies were corroborated in some but not confirmed in other studies. Attempts to replicate associations of genetic factors with lifespan detected in earlier studies using the Genome Wide Association Studies (GWAS) were not very successful: none or a very small fraction of the previously detected associations have been confirmed. The genetic variants detected in GWA studies of complex traits, including lifespan, explain only a small portion of the narrow sense heritability that was calculated for such traits in the pre-genomic era. This situation requires an explanation and indicates the need for developing new approaches to the analysis of genetics of aging and lifespan. We discuss one such approach, which combines GWAS of human lifespan with analyses of longitudinal data on changes in health status and physiological state. The approach addresses the issue of “missing heritability“ and allows for studying joint influence of many small-effect genetic variants on lifespan and other durations (e.g., free of major diseases and disability lifespan) by constructing polygenic score indices and investigating their influence on such traits. It also allows for investigating differences in age trajectories of physiological indices, as well as health histories for individuals with different genetic background. We also show why conventionally used strategy to confirm GWAS research findings often fails to do so.

**Vadim Gladyshev** (Brigham and Women's Hospital, Harvard University Medical School, USA) presented “Exceptional longevity of the naked mole rat: insights from genome sequencing and transcriptomic analysis”. The naked mole rat (NMR, Heterocephalus glaber) is a strictly subterranean, extraordinarily long-lived eusocial mammal. Although the size of a mouse, its maximum lifespan exceeds 30 years and makes this animal the longest living rodent. NMRs show negligible senescence, no age-related increase in mortality, and high fecundity until death. In addition to delayed aging, NMRs are resistant to both spontaneous cancer and experimentally induced tumorigenesis. NMRs pose a challenge to the theories that link aging, cancer and redox homeostasis. Although characterized by significant oxidative stress, the NMR proteome does not show age-related susceptibility to oxidative damage nor increased ubiquitination. NMRs naturally reside in large colonies with a single breeding female, the “queen,” who suppresses the sexual maturity of her subordinates. NMRs also live in full darkness, at low oxygen and high carbon dioxide concentrations, and are unable to sustain thermogenesis nor feel certain types of pain. We report sequencing and analysis of the NMR genome, which revealed unique genome features and molecular adaptations consistent with cancer resistance, poikilothermy, hairlessness, altered visual function, circadian rhythms and taste sensing, and insensitivity to low oxygen. This information provides insights into NMR's exceptional longevity and capabilities to live in hostile conditions, in the dark and at low oxygen. The extreme traits of NMR, together with the reported genome and transcriptome information, offer unprecedented opportunities for understanding aging and advancing many other areas of biological and biomedical research.

**Yelena Budovskaya** (University of Amsterdam, Holland) presented “Molecular aging driven by Wnt signaling in *C. elegans*.” We are using a system biology approach to reveal the molecular basis for aging in nematodes *C. elegans* by characterizing gene expression differences between young and old animals and then determining at a molecular level how these changes contribute to aging. We used DNA microarrays to profile gene expression changes associated with aging. This analysis revealed that gene expression differences between young and old animals are under control of a relatively simple gene regulatory network that involves the *elt-3*, *elt-5*, and *elt-6* GATA transcription factors. Expression of *elt-5* and *elt-6* increases in old age, leading to decreased expression of *elt-3*, thus causing changes in the expression of the many downstream target genes. We found no evidence that it is caused by cellular damage or environmental stresses. Rather, we found that *elt-3* expression in the adult is controlled by increased expression of the repressors *elt-5* and *elt-6*, which also guide *elt-3* expression during development. These results suggest that age-regulation of *elt-3* is caused by age-related drift of an intrinsic developmental program that becomes imbalanced in old age. This pathway, which is one of the first and clearest examples of developmental drift, may in part be responsible for some of the age-related changes that occur as the worm grows old. A key unanswered question is what causes *elt-3/elt-5/elt-6* transcriptional drift during aging? In *C. elegans*, Wnt/Wingless signaling pathways activate *elt-5* and *elt-6* expression during development. Here we demonstrated that Wnt signaling is responsible for the increased expression of *elt-5* and *elt-6* GATA transcription factors during aging. Mutations in the components of the “Wnt/b-catenin asymmetry pathway”, such us *mom-2*/Wnt, the β-catenin, *wrm-1*, or *pop-1*/TCF decreases expression levels of the *elt-5* and *elt-6* GATA transcription factors throughout life, leading to increased *elt-3* GATA expression. We demonstrated that inactivation of the “Wnt/b-catenin asymmetry pathway” starting at the firtst day of adulthood, extends lifespan of otherwise wild type animals by ~40 - 50%. These results indicate that changes in Wnt signaling – a regulator of the *elt-3/elt-5/elt-6* GATA transcriptional circuit during normal development – plays an important role in the age-related regulation of the *elt-3/elt-5/elt-6* transcriptional circuit and possibly many others.

**Yousin Suh** (Einstein College of Medicine, USA) presented “MicroRNA, aging, and longevity”. Expression levels of miRNAs in lymphoblastoid cell lines and plasma are associated with longevity in humans and that some of these miRNAs are known to target conserved pathways of aging, including the IGF signaling pathway.

**Alex Maslov** (Einstein College of Medicine, USA) presented “DNA damage in normal and premature aging: impact on the aging epigenome”. Quantitative long-range PCR is a reliable method for quantitative assessment of DNA damageю Normally, aged animals demonstrate an increased level of DNA damage across the genome.

**Julia Hoffmann** and **Thomas Roeder** (University of Kiel, Germany) reported that mild *sir2* overexpression in the fat body of *D. melanogaster* extends life span and reduces relative body fat content in both males and females. The response to dietary restriction (DR) is gender-specific: DR reduces relative body fat content in females but increases it in males. Regulated gene sets of males and females show a very small overlap.

## CONFERENCE PICTURES

**Figure:**
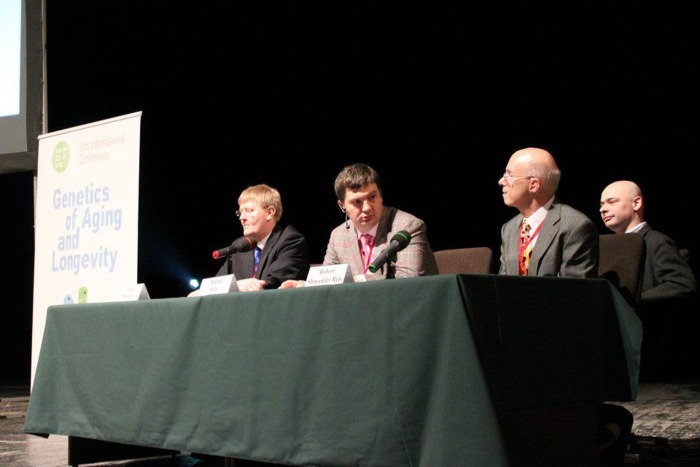
Alexey Moskalev (co-chair of the Organizing committee), Mikhail A. Batin (co-chair of the Organizing committee) and Robert Shmookler Reis

**Figure:**
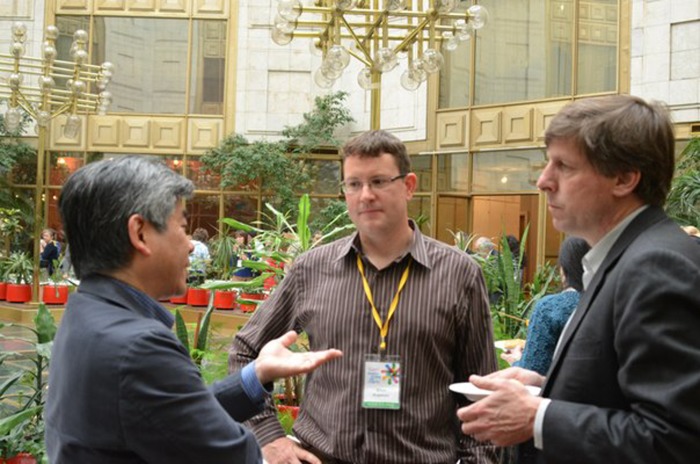
Richard Morimoto, Brian Kraemer and Brian Kennedy

**Figure:**
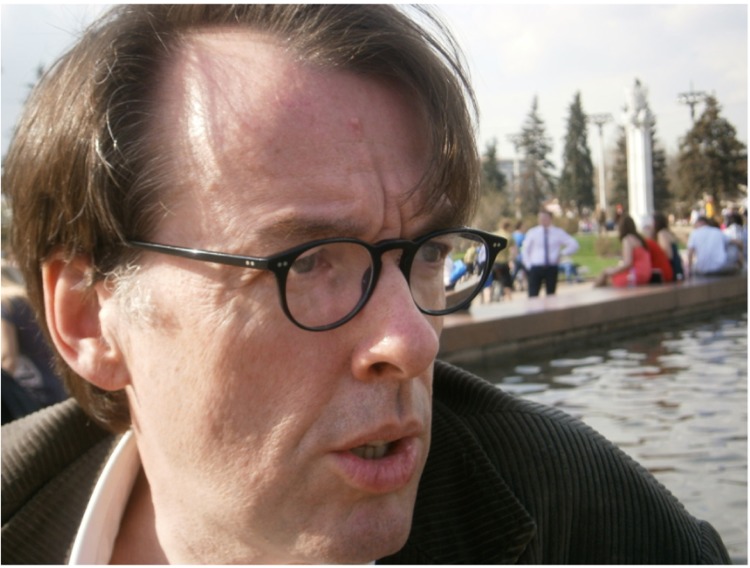
David Gems

**Figure:**
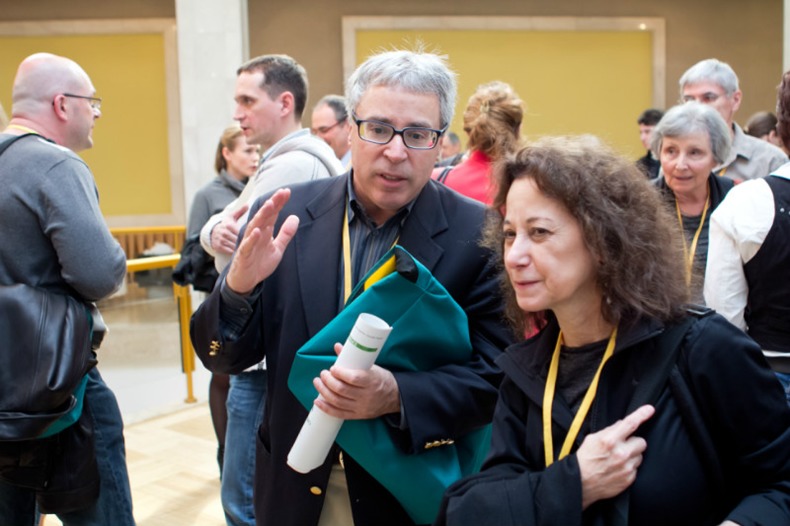
Nir Barzilai and Judith Campisi

**Figure:**
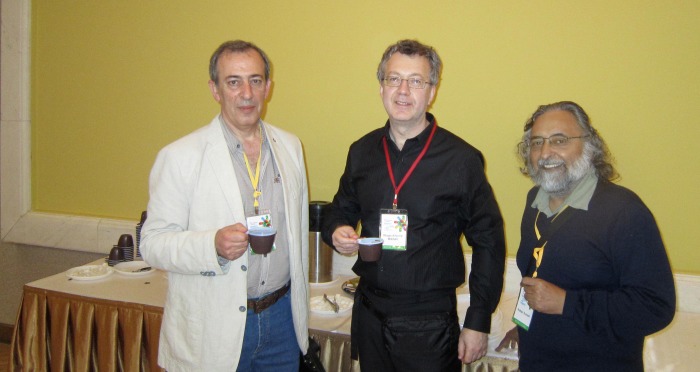
Vadim Fraifeld, Mikhail V. Blagosklonny and Suresh Rattan

**Figure:**
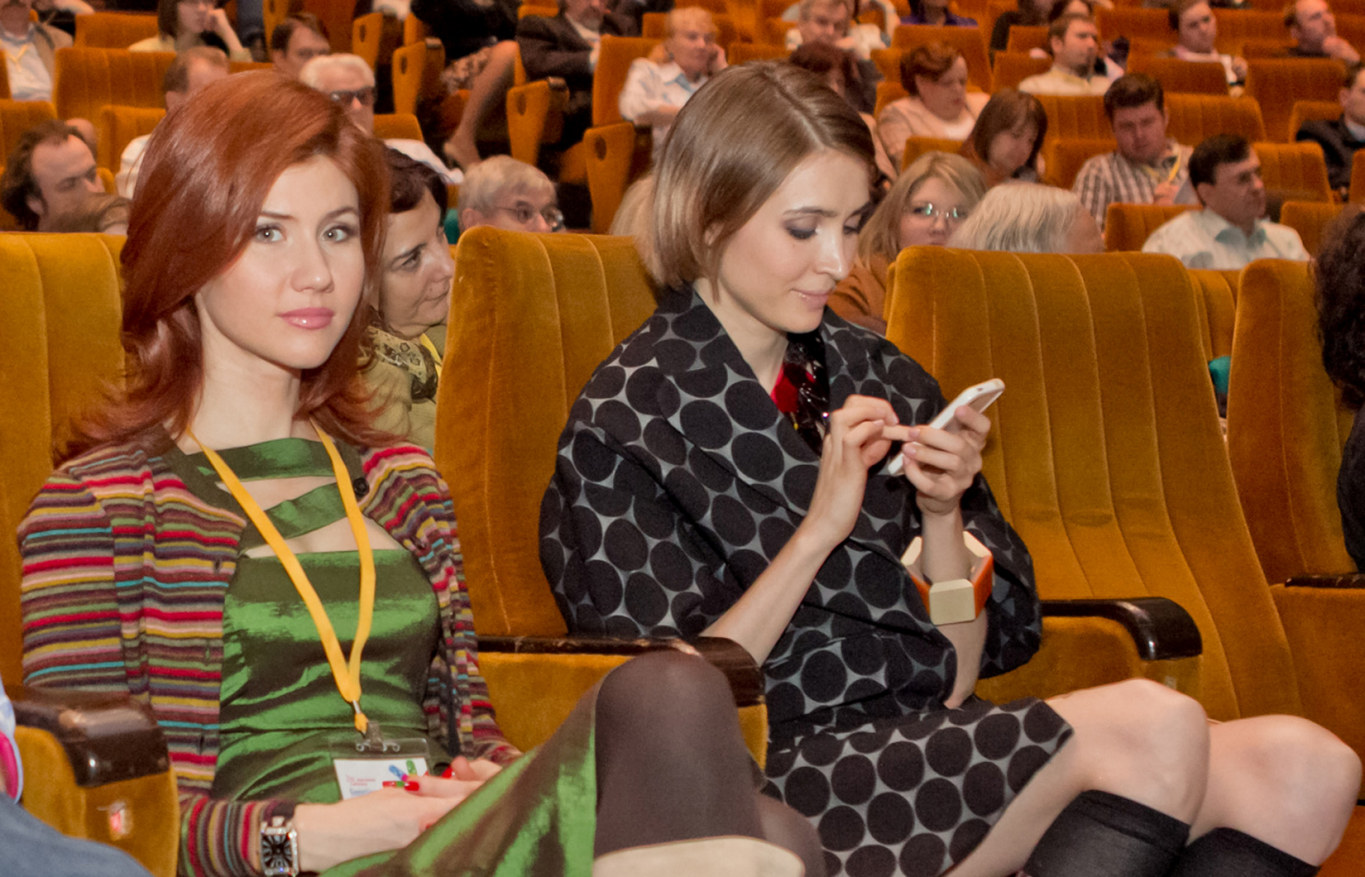
Anna Chapman (President of the Fund UMA, non-profit supporting young scientists) and Maria Konovalenko (Conference secretariat)

